# Modeling neuropsychiatric disorders using human induced pluripotent stem cells

**DOI:** 10.1007/s13238-019-0638-8

**Published:** 2019-05-27

**Authors:** Meiyan Wang, Lei Zhang, Fred H. Gage

**Affiliations:** 1 Laboratory of Genetics, The Salk Institute for Biological Studies, 10010 N. Torrey Pines Rd, La Jolla, CA 92037 USA; 2 Neurobiology Section, Division of Biological Sciences, University of California San Diego, La Jolla, CA 92093 USA

**Keywords:** neuropsychiatric disorders, iPSCs, brain organoid, schizophrenia, autism spectrum disorder, bipolar disorder

## Abstract

Neuropsychiatric disorders are complex disorders characterized by heterogeneous genetic variations, variable symptoms, and widespread changes in anatomical pathology. In the context of neuropsychiatric disorders, limited access to relevant tissue types presents challenges for understanding disease etiology and developing effective treatments. Induced pluripotent stem cells (iPSCs) reprogrammed from patient somatic cells offer an opportunity to recapitulate disease development in relevant cell types, and they provide novel approaches for understanding disease mechanisms and for development of effective treatments. Here we review recent progress and challenges in differentiation paradigms for generating disease-relevant cells and recent studies of neuropsychiatric disorders using human pluripotent stem cell (hPSC) models where cellular phenotypes linked to disease have been reported. The use of iPSC-based disease models holds great promise for understanding disease mechanisms and supporting discovery of effective treatments.

## INTRODUCTION

The recent advances in technologies that enable adult human somatic cells to be reprogrammed into iPSCs (Takahashi et al., 2007; Yu et al., 2007) hold enormous potential in the field of regenerative medicine (Shi et al., 2017). Despite the promise of iPSCs as an autologous cell source for cell transplantation therapy, concerns have been raised regarding the clinical applications of iPSCs (Gore et al., 2011; Zhao et al., 2011, 2015b; Ji et al., 2012; Kang et al., 2016). While much work remains to be done to improve the safety and reliability of the technology, iPSCs have provided a unique opportunity to study developmental neuropsychiatric diseases. In the past, this type of research has have been challenging due to the inaccessibility of relevant live tissues and cell types. The lack of reliable models has significantly hindered the progress in understanding the pathogenesis of the disease and development of novel treatments.

Animal models provide valuable insights into the mechanisms of specific functions of genes. However, modeling of human neuropsychiatric disorders in animals is extremely challenging given the complexity of the disorders, the subjective nature of many symptoms, and the lack of biomarkers and reliable tests (Nestler and Hyman, 2010). The differences in the brain structures of human and mouse have also made it difficult to study behaviors related to higher-function brain areas such as the dorsolateral prefrontal cortex, one of the most recently derived parts of the human brain that is often implicated in neuropsychiatric disorders (Tekin and Cummings, 2002). In addition, it is difficult to assess some complex phenotypes in mouse, such as psychosis.

Postmortem human brains serve as valuable sources for examining pathological changes in patients. However, these tissues only represent the disease endpoint, whereas the alterations that lead to the development of the diseases often occur early in development. Postmortem brain studies are thus limited in their ability to reveal dynamic cellular changes that are often important in the disease mechanisms.

iPSC technology has opened the field to new discoveries in neuropsychiatric diseases (see Table 1 for selected studies). iPSCs can be directly generated from somatic cells by the introduction of reprogramming factors (Takahashi et al., 2007). Moreover, iPSCs are capable of differentiating into adult cell types through directed differentiation with a combination of small molecules, growth factors, and morphogens, allowing the derivation of unlimited disease-relevant cells carrying the variations that caused or facilitated the development of the disease. These cells include human neurons, a previously inaccessible cell type.

**Table 1:** Selected studies investigating neuropsychiatric disorders using hiPSCs from patients.

Disease	Type of cells	Genetic variants	Phenotypes	Reference
Rett syndrome	Neurons	*MECP2*	Fewer synapses, reduced spine density, smaller soma size, altered calcium signaling, electrophysiological defects	(Marchetto et al., 2010)
Rett syndrome/ASD	Neurons	*CDKL5*	Aberrant dendritic spines	(Ricciardi et al., 2012)
Rett syndrome	NPCs	*MECP2*	Migration defects in layered 3D culture; reduced neurite outgrowth and fewer synapses	(Zhang et al., 2016)
Non-syndromic ASD	Neurons	*TRPC6*	Altered neuronal development, morphology and function	(Griesi-Oliveira et al., 2015)
ASD	Organoids	Idiopathic	Accelerated cell cycle, overproduction of GABAergic inhibitory neurons	(Mariani et al., 2015)
ASD	NPCs, Neurons	Idiopathic	Increased cell proliferation of NPCs, abnormal neurogenesis, reduced synaptogenesis, defects in neuronal networks	(Marchetto et al., 2017)
ASD	Neurons	16p11.2 deletion/ 16p11.2 duplication	Increased soma size and dendrite length in 16pdel neurons; reduced neuronal size and dendrite length in 16pdup neurons	(Deshpande et al., 2017)
ASD	Cortical neurons	Idiopathic	Morphological growth acceleration of early neuron development	(Schafer et al., 2019)
Timothy syndrome	Cortical neurons	*CACNA1C*	Abnormal expression of tyrosine hydroxylase and increased production of norepinephrine and dopamine	(Pasca et al., 2011)
Timothy syndrome	Subdomain specific forebrain spheroids	*CACNA1C*	Abnormal migratory saltations	(Birey et al., 2017)
Fragile X syndrome	Neurons	*FMR1*	Abnormal homeostatic synaptic plasticity	(Zhang et al., 2018)
Bipolar disorder	NPCs	Idiopathic	Increased CXCR4 expression; phenotypic differences in neurogenesis	(Madison et al., 2015)
Bipolar disorder	DG neurons	Idiopathic	Mitochondrial abnormalities, hyperactive action-potential firing	(Mertens et al., 2015)
Bipolar disorder	Neurons	Idiopathic	Increased expression of miR-34a	(Bavamian et al., 2015)
Bipolar disorder	DG neurons	Idiopathic	Hyperactive action-potential firing, fast after-hyperpolarization	(Stern et al., 2018)
SZ	Neurons	Idiopathic	Diminished neuronal connectivity, decreased neurite number	(Brennand et al., 2011)
SZ	DG neurons	Idiopathic	Deficits in the generation of DG neurons, reduced neuronal activity, reduced spontaneous neurotransmitter release	(Yu et al., 2014)
SZ	NPCs	15q11.2 microdeletion	Deficits in adherens junctions and apical polarity	(Yoon et al., 2014)
SZ	Neurons	Idiopathic	Elevated levels of dopamine, norepinephrine, and epinephrine secretion	(Hook et al., 2014)
SZ	Neurons	22q11 deletion	Increase L1 copy number	(Bundo et al., 2014)
SZ	NPCs	Idiopathic	Higher variability in the levels of HSF1 activation induced by environmental challenges	(Hashimoto-Torii et al., 2014)
SZ	Forebrain neurons	*DISC1*	Synaptic vesicle release deficits	(Wen et al., 2014)
SZ	NPCs	Idiopathic	Abnormal gene expression and protein levels related to cytoskeletal remodeling and oxidative stress	(Brennand et al., 2015)
SZ	Neurons	22q11.2 microdeletion	45 differentially expressed miRNAs	(Zhao et al., 2015a)
SZ	NPCs	*DISC1*	Increased miR-219 expression	(Murai et al., 2016)
SZ	Glial progenitors/humanized glial chimeric mice	Idiopathic	Premature migration into the cortex; delayed astrocytic differentiation and abnormal astrocytic morphologies	(Windrem et al., 2017)
SZ	CA3 neurons, DG neurons	Idiopathic	Reduced activity in DG-CA3 co-culture, deficits in spontaneous and evoked activity in CA3 neurons	(Sarkar et al., 2018)
SZ	Cortical interneurons	Idiopathic	Defects in synaptic density and arborization	(Shao et al., 2019)
SZ	Microglia and neurons	Idiopathic	Increased synapse elimination in neural cultures and isolated synaptosomes	(Sellgren et al., 2019)
AxD	Astrocytes and OPCs	*GFAP*	GFAP aggregates and Rosenthal fibers; AxD astrocytes inhibit OPC proliferation and myelination	(Li et al., 2018)
AxD	Astrocytes	*GFAP*	GFAP aggregates; impaired extracellular ATP release; enlarged and heterogeneous morphology coupled with perinuclear localization of endoplasmic reticulum and lysosomes	(Jones et al., 2018)
AxD	Astrocytes	*GFAP*	Increased expression of A-type lamin and nuclear yes-associated protein; increased numbers of stress fibers in the soma of AxD astrocytes	(Wang et al., 2018)
Phelan-McDermid syndrome	Neurons	22q13 deletion	Major defects in excitatory but not inhibitory synaptic transmission	(Shcheglovitov et al., 2013)
Major depressive disorder	Forebrain neurons	Idiopathic	Serotonin-induced hyperactivity downstream of upregulated excitatory serotonergic receptors in selective serotonin reuptake inhibitors-nonremitters	(Vadodaria et al., 2019)

Most neuropsychiatric diseases, including autism spectrum disorder (ASD), schizophrenia (SZ), bipolar disorder, and major depression, have a strong but complex genetic component. Multiple low penetrance genetic variants contribute to the etiology of those disorders (Schizophrenia Working Group of the Psychiatric Genomics, 2014; Bipolar et al., 2018; Wray et al., 2018; Grove et al., 2019). Because human iPSCs (hiPSCs) capture the genetic diversity of the patient, they are particularly useful for modeling how complex genetic variants lead to the pathogenesis of the disease.

## NEURAL SPECIFICATION FROM HUMAN PLURIPOTENT STEM CELLS

The mammalian brain is composed of many cell populations that differ in their molecular, morphological, connectional, and functional properties. The recent advances in single cell technologies have enabled the identification of new neuronal subtypes in both mouse and human brains (Darmanis et al., 2015; Zeisel et al., 2015; Lake et al., 2016), offering new opportunities to study the diversity of the building blocks of the mammalian brain. However, how distinct subpopulations of cells respond to perturbation and how diseased brains differ in their molecular and functional signatures remain to be explored.

Substantial progress has been made in directing hPSCs differentiating into regional and functional specific neuronal subtypes. These differentiation protocols are often based upon key molecular signals in neural commitment and regionalization identified in animal models. Manipulations of the principles in early neural development have enabled the generation of highly enriched subpopulations of neurons and glial cells *in vitro* with functional aspects that resemble those in the brain (Mertens et al., 2016).

At the gastrulation stage, nervous system specification initiates from ectoderm cells, in a process triggered by molecules that inhibit bone morphogenetic protein (BMP) signaling and/or activate the fibroblast growth factor (FGF) pathways (Edlund and Jessell, 1999). The neural plate then folds to form the neural tube, from which the brain and spinal cord develop. Specification of subtypes of neural progenitor cells (NPCs) in the neural tube is achieved by the coordinated temporal and spatial gradient of morphogens and mitogens. For example, sonic hedgehog (SHH) is secreted from the notochord and floor plate of the ventral neural tube, whereas Wnt and BMP are secreted from the roof plate in the dorsal neural tube (Ciani and Salinas, 2005; Liu and Niswander, 2005; Fuccillo et al., 2006). This morphogen gradient specifies the neural subtypes along the dorsal-ventral (D-V) axis. The differentiation of the neural tube along the anterior-posterior (A-P) axis is coordinated by the gradient of timely and spatially regulated factors, including FGFs and retinoic acid (RA) (Maden, 2007; Mason, 2007; Guillemot and Zimmer, 2011).

Following this principle, hPSCs are first switched from the self-renewing condition to promote differentiation toward all three germ layers by the removal of self-renewal growth factors. Without extrinsic cues, the default differentiation is towards forebrain identity (Munoz-Sanjuan and Brivanlou, 2002). Coordinating morphogens along both the A-P axis and D-V axis is used for patterning of NPC subtypes (Tao and Zhang, 2016). Regionalized NPCs can be turned into specific neuronal subtypes through differentiation induction and neurotrophic support. For example, the progenitor cells with the dorsal forebrain identity become glutamatergic neurons, whereas the ventral progenitors become gamma-aminobutyric acid-ergic (GABAergic) or cholinergic neurons.

### Specification of cortical excitatory neurons

Cortical excitatory neurons are born in the dorsal forebrain *in vivo*. The telencephalic/forebrain identity develops in the absence of any extrinsic cues and is enhanced by the inhibition of morphogens such as Wnts or BMPs (Wilson and Houart, 2004; Gaspard et al., 2008). The telecephalon then undergoes patterning along the D-V axis, from which cortical excitatory neural precursors are generated in the dorsal telecephalon (Sur and Rubenstein, 2005). The characteristics of corticogenesis are recapitulated in directed differentiation from hPSCs (Watanabe et al., 2005; Eiraku et al., 2008; Gaspard et al., 2008; Chambers et al., 2009; Shi et al., 2012). The layer-identity of a cortical neuron is linked to the timing of its generation: deep layer cortical neurons are generated first, followed by upper layer neurons. Analysis of a recent differentiation protocol has demonstrated that the *in vitro* generation of excitatory cortical neurons recapitulates the layer-specific sequential order (Espuny-Camacho et al., 2013). The proportion of each layer-specific neuronal subtype depends on differentiation conditions (Gaspard et al., 2008; Espuny-Camacho et al., 2013). Outer radial glial cells, in a region that has undergone significant evolutionary changes in cortical neurogenesis, have also been detected in cortical differentiation from hPSCs (Shi et al., 2012) but not from mouse PSCs (Lancaster et al., 2013). Alterations in corticogenesis have been implicated in many developmental neuropsychiatric disorders. Regional patterns of gene expression that typically differ in frontal and temporal cortex are significantly attenuated in the ASD brain based on transcriptomic analysis (Voineagu et al., 2011). Abnormal neurogenesis leading to functional defects in neuronal networks has also been reported using NPCs from iPSCs reprogrammed from ASD individuals. Interestingly, a drug currently in clinical trials could rescue defects in neuronal networks for ASD *in vitro* (Marchetto et al., 2017). In a recent study recapitulating cortical development from ASD patients-derived iPSCs, ASD-associated changes in the maturational sequence of early neuron development have been reported (Schafer et al., 2019). Temporal dysregulation of specific gene networks is tracked back to a pathologically primed stage in neural stem cells. Interestingly, circumventing the neural progenitor stage by direct conversion of ASD iPSCs into induced neurons abolished the ASD-associated phenotypes. These findings together support the idea that aberrant early cortical development contributes to the emergence of ASD.

### Specification of hippocampal neurons

The hippocampus arises from the caudomedial edge of the dorsal telencephalic neuroepithelium adjacent to the cortical hem (Lee et al., 2000). To generate NPCs with hippocampal identity, embryoid bodies (EBs) (aggregates obtained from hPSC cultures) are treated with antagonists of the SHH pathway and a cocktail of factors mimicking the patterning of the forebrain, including Dickkopf-related protein 1 (DKK1), Noggin, and transforming growth factor-β (TGF-β) inhibitor SB431542, to instruct the dorsal telencephalic precursor identity (Yu et al., 2014). The hippocampal patterned NPCs express genes in early stages of hippocampal neurogenesis, such as SOX2, PAX6, EMX2 and FOXG1. The cortical hem provides a Wnt source to the progenitor cells (Lee et al., 2000). In the presence of high concentrations of Wnt3a, hippocampal dentate gyrus (DG)-like granule neurons that express prospero homeobox protein 1 (PROX1) can be generated from hippocampal NPCs (Yu et al., 2014). Hippocampal DG neurons generated from SZ patient iPSCs exhibited deficits in the generation of DG granule neurons, reduced neuronal activity, and reduced levels of spontaneous neurotransmitter release (Yu et al., 2014). Hippocampal DG neurons differentiated from bipolar patient iPSCs showed hyperexcitability in eletrophysiological recordings (Mertens et al., 2015; Stern et al., 2018). This hyperexcitability phenotype of young neurons in bipolar disorder can be selectively reversed by lithium treatment only in the neurons derived from patients who responded to lithium treatment (Mertens et al., 2015; Stern et al., 2018). The hippocampus proper is defined by the DG and cornu ammonis (CA). An efficient differentiation paradigm that generates multiple CA3 pyramidal neuron subtypes has also been developed using the same hippocampal patterned NPCs (Sarkar et al., 2018). During development, Wnt regulates cell proliferation and specification of the DG and CA fields of the hippocampus (Galceran et al., 2000; Lee et al., 2000). DG primordia locate next to the cortical hem whereas CA3 primodia locate more dorsally. To induce CA3 pyramidal neuron fate, hippocampal NPCs were exposed to lower amount of Wnt3a compared to the DG differentiation protocol (Sarkar et al., 2018). This differentiation paradigm produced several CA3 neuron subtypes, including a human specific SCGN-expressing CA3 neuron subtype.

### Specification of inhibitory neurons

*In vivo*, GABAergic interneurons within the telencephalon populate from one of two embryonic subcortical progenitor zones, the medial and caudal ganglionic eminences (MGE and CGE) (Wonders and Anderson, 2006; Kelsom and Lu, 2013). The interneurons arising from each progenitor zone represent complementary interneuron subtypes (Kepecs and Fishell, 2014). Within the cortex, parvalbumin (PV) fast spiking interneurons and somatostatin interneurons arise from the MGE, whereas neurogliaform, bipolar, and VIP multipolar interneurons arise from the CGE (Lee et al., 2010; Miyoshi et al., 2010; Kepecs and Fishell, 2014). Several groups were able to generate MGE-like progenitors using small molecules that inhibit Wnt and SMAD signaling coupled with timely SHH exposure to induce ventral telencepahlic fate (Liu et al., 2013; Maroof et al., 2013; Nicholas et al., 2013). In culture, the MGE-like cells take a remarkably long time to develop into GABAergic interneuron subtypes with mature physiological properties, mimicking human neural development *in vivo* (Nicholas et al., 2013). A recent single cell study of the cortical inhibitory interneuron development and diversification has revealed that transcription factor MEF2C, a gene linked to neuropsychiatric and neurodevelopmental disorders, plays an important role in early precursors of PV-expressing interneurons (Mayer et al., 2018). Cortical interneurons generated from iPSCs derived from SZ subjects had dysregulated expression of protocadherin genes, which lie within documented SZ loci (Shao et al., 2019). SZ cortical interneurons showed defects in synaptic density and arborization that could be reversed by inhibitors of a downstream kinase in the protocadherin pathway. Similar phenotype is observed in mice lacking protocadherin-α. These findings reveal an intrinsic abnormality in SZ cortical interneurons, providing evidence that dysregulation of interneuron specification and development is linked to neuropsychiatric disorders.

### Specification of microglia

Microglia is the resident macrophages of the central nervous system (CNS) and function in inflammation and the clearance of dead cells and debris. Microglia also play an important role in development and homeostasis, raising an interesting possibility that microglia dysfunction could contribute to neurological and psychiatric diseases. Neural networks are being constantly modified by the elimination and strengthening of the synapses. During late adolescence and early adulthood, synaptic pruning by microglia occurs to remove the excess synapses generated during development and make the brain connections more streamlined. Feinberg suggested that improper synaptic pruning during this period could lead to the development of SZ (Feinberg, 1982).

Microglia originate almost exclusively from erythromyeloid progenitors (EMP) generated during primitive hematopoiesis from yolk-sac cells (Ginhoux et al., 2010; Schulz et al., 2012; Kierdorf et al., 2013). EMPs further develop and migrate into the developing neural tube shortly before the closure of the blood-brain barrier. Microglia progenitors then mature in the CNS and facilitate CNS development. Recent murine studies identified key cytokines and transcription factors required for microglia development and maintenance *in vivo*, including IL-34, colony-stimulating factor1, and TGF-β (Greter et al., 2012; Wang et al., 2012; Butovsky et al., 2014; Yamasaki et al., 2014). A few studies have succeeded in generating microglia-like cells from hPSCs. These protocols all started with generating primitive hematopoietic cells and then differentiating those cells into microglia, recapitulating environmental cues present in the developing embryo (Muffat et al., 2016; Abud et al., 2017; Pandya et al., 2017). A human embryonic stem cell (hESC) model of Rett syndrome has indicated smaller primary microglial-like cells than their isogenic controls (Muffat et al., 2016). In an *in vitro* model that applied cellular reprogramming to study SZ-specific microglia-mediated synapse engulfment, excessive pruning was also detected in induced microglia-like cells generated from SZ patients (Sellgren et al., 2019).

### Specification of astrocytes

Astrocytes are a major component of the human CNS and provide metabolic and trophic support to neurons. Astrocytes play an essential role in establishing and regulating neural circuits and have been implicated in many aspects of neurodevelopment and in neurodevelopmental diseases (Khakh and Sofroniew, 2015). During embryonic development, astrocytes are generated from radial glial cells at the subventricular zone (SVZ). Radial glial cells give birth to neurons in early embryonic development and to glial cells in the late phase and postnatal period. This neurogenesis-to-gliogenesis switch requires Notch signaling, which activates the JAK/STAT3 pathway and promotes gliogenesis (Morrison et al., 2000). The generation of astrocytes from hPSCs recapitulates the gliogenic switch during embryonic development. Glial progenitor differentiation from hPSCs requires neural induction, coupled with an extended time in culture for the gliogenic switch. To differentiate glial progenitors to astrocytes, BMP and ciliary neurotrophic factor are added to activate the JAK/STAT3 pathway (Krencik et al., 2011). Functional maturation of astrocytes can take up to six months (Krencik and Zhang, 2011; Krencik et al., 2011). An efficient protocol to derive functional astrocytes from hPSCs has been described (Santos et al., 2017), in which the glial progenitor cells can be propagated, expanded and frozen as intermediates. hPSCs are first converted into NPCs and then patterned to glial progenitors that could later give rise to functional astrocytes. Neural induction was achieved in EBs by SMAD inhibition. To induce JAK/STAT3 signaling, PDGF was added. The patterned glial progenitors expressed Nestin, A2B5, a cell surface ganglioside in astrocyte precursors, and nuclear factor IA, a transcription factor activated by Notch, which is required for gliogenesis. To induce differentiation and maturation of astrocytes, leukemia inhibitory factor was used in serum-free condition in neuronal medium. Astrocytic markers can be detected after four to six weeks of differentiation, such as CD44, S100 calcium-binding protein (S100β), glial fibrillary acidic protein (GFAP), and aldehyde dehydrogenase 1 family member L1, an early marker of astrocyte maturation. The hPSC-derived astrocytes are capable of glutamate uptake and display calcium transients. Recent studies of Alexander disease (AxD) have identified GFAP aggregates and Rosenthal fibers in AxD patient iPSC-derived astrocytes (Jones et al., 2018; Li et al., 2018). Moreover, these iPSC-based models also revealed that AxD astrocytes inhibited proliferation of human iPSC-derived oligodendrocyte progenitor cells (OPCs) in co-culture and reduced their myelination potential (Li et al., 2018), recapitulating the pathological hallmark of AxD.

### Specification of oligodendrocytes

Recent evidence has suggested that there are abnormalities in oligodendrocyte function in psychiatric conditions (Hakak et al., 2001; Tkachev et al., 2003; Nave and Ehrenreich, 2014). Oligodendrocytes arise late during development, providing support and insulation to axons in the CNS. Oligodendrocytes originate from glial-restricted progenitor cells, known as OPCs, which are generated by subventricular cells in the developing brain and spinal cord (Emery, 2010). OPCs divide and migrate throughout the CNS (Emery, 2010). The process of oligodendrocyte differentiation from hPSCs essentially follows the well-characterized developmental origin of OPCs. Specification of OPCs starts with neural induction, and then followed by SHH activation. Oligodendrocytes generated from hPSCs are capable of producing myelin sheaths when co-cultured with neurons or transplanted into the mouse/rat brain or spinal cord (Keirstead et al., 2005; Nistor et al., 2005; Hu et al., 2009; Kerr et al., 2010; Sharp et al., 2010; Wang et al., 2013; Piao et al., 2015; Rodrigues et al., 2017). Direct conversion of OPCs from fibroblasts and induction of oligodendrocytes in human cortical spheroids have also been reported (Yang et al., 2013; Madhavan et al., 2018).

### Functional circuits

Neurons are organized into circuits in neural networks to perform information processing and transmission. Dysregulated synaptic function has been hypothesized to underlie the altered neuronal function in complex neuropsychiatric disorders. It is therefore imperative to generate an *in vitro* model where synaptic function can be directly assessed. Neuronal maturation has been limited *in vitro* due to the difficulties of maintaining a long-term culture required for neurons to mature while still keeping them in a healthy condition. Substantial improvements in these areas have been made in the past decade. Synaptic markers such as synapsin and postsynaptic density protein 95 (PSD-95) can be detected in hPSCs-derived neural cultures. Moreover, spontaneous synaptic currents have been detected by electrophysiological recordings, indicating the formation of active neural networks. These advances have led to the discoveries of aberrant synaptic functions in iPSC models of SZ. In iPSCs derived from idiopathic SZ patients, diminished neuronal connectivity has been reported in conjunction with decreased neurite number (Brennand et al., 2011). A more recent study of iPSCs derived from SZ patients carrying a four base-pair deletion in Disrupted-in-schizophrenia 1 (*DISC1*), a gene that co-segregates with major psychiatric disorders, has reported aberrant synaptic formation and synaptic vesicle release deficits in patient-derived neurons (Wen et al., 2014).

Establishing a functional neural circuit *in vitro* using hPSC-derived neurons will help decipher the abnormalities at the circuit level in disease conditions. This is especially important for modeling neuropsychiatric disorders in which the imbalanced/abnormal neural networks have been implicated in the disease etiology. To recapitulate the disease status *in vitro*, it is also important to study the functional circuits in which the disease has been implicated. Attempts to model neuronal pathology of the cells with the functional targets have been made. Hippocampal circuitry has been studied using hPSC-derived DG and CA3 neurons (Sarkar et al., 2018). Microfluidic devices, involving a two-compartment system connected by narrow grooves, have been used to reconstruct DG-CA3 circuitry in which DG neurons are cultured in one compartment and CA3 neurons are cultured in the other compartment. In this system, axonal growth is allowed through the narrow grooves connecting the two compartments whereas cell migration is restricted. Rabies virus infection of the CA3 neurons permits the detection of presynaptic neurons that monosynaptically connect to the postsynaptic CA3 neurons. The presence of the DG-CA3 connection suggests that microfluidic device-based reconstruction of circuitry *in vitro* mimics the synaptic connections *in vivo*. Hippocampus-dependent functional impairment has been reported in SZ (Cirillo and Seidman, 2003; Freedman and Goldowitz, 2010; Tamminga et al., 2010; Rasetti et al., 2014). In postmortem studies, reduced glutamate transmissions in the mossy fiber pathway have also been indicated in SZ (Kolomeets et al., 2007; Tamminga et al., 2010; Li et al., 2015). Intriguingly, co-culture of the DG and CA3 neurons derived from SZ iPSCs has revealed decreased spontaneous activity (Sarkar et al., 2018). All together, these studies indeed suggest great promise in studying defects in transmission in neurons generated from patients with mental disorders.

## A THREE-DIMENSIONAL (3D) BRAIN ORGANOID MODEL

Monolayer neuronal cultures often generate highly enriched uniform populations of targeted cell types (Pasca, 2018). The scalability and simplicity of two-dimensional (2D) cultures make the system more suitable for mechanistic studies and large-scale screening, e.g., CRISPR-Cas9 screening or high-throughput drug testing. However, the complex nature of brain tissues requires a better system to recapitulate the organogenesis, which involves local proliferation and patterning, cell fate determination, complex cell–cell interactions, and long-distance migration. Promisingly, 3D neural cultures, including brain organoids and neural spheroids, may serve this purpose.

Compared to 2D culture, in which cells are purified and isolated, 3D culture allows cells of different types to interact. However, understanding the cellular composition of brain organoids is challenging. To solve this issue, single-cell RNA sequencing allows systematic interrogation of many genes from large number of cells, thereby offering a good opportunity to isolate distinct cell clusters and study their molecular properties. A diversity of cell types, including NPCs, OPCs, glia, neurons, and retinal cells, has been identified in brain organoids (Quadrato et al., 2017). Within each cell type, subtypes can be further classified. For instance, 30 transcriptionally distinct subclusters can be identified in a neuronal cluster (Quadrato et al., 2017), and glial cells can be specified into at least three clusters (Sloan et al., 2017). Thus, endogenous cellular diversity can be recapitulated in organoids, and chances are that human-specific cell types that are transiently present in certain developmental stages might be discovered (Amiri et al., 2018).

The 3D organoid is far beyond a mixture of diverse cell types, but rather a self-assembly of cells under temporal and spatial regulation. In early organoid cultures, neural stem cells or radial glia reside next to the ventricle-like structure and give birth to neurons in the outer layers (Kadoshima et al., 2013; Lancaster et al., 2013; Pasca et al., 2015; Qian et al., 2016; Bershteyn et al., 2017). Astrocytes are rarely observed until six months or later (Lancaster et al., 2013; Pasca et al., 2015; Qian et al., 2016; Sloan et al., 2017). Oligodendrocytes can be induced by exogenous molecules, including PDGF-AA, IGF-1 (D50-60), T3, and PERK inhibitor, in the late stage of organoids culture (Madhavan et al., 2018). This timeline is consistent with early brain development, where neurogenesis starts prenatally and is followed by the gliogenesis postnatally (Miller and Gauthier, 2007). A recent study has confirmed the feasibility of using organoids to study molecular and cellular events in early cortical development (Amiri et al., 2018). Forebrain organoids were patterned and maintained in differentiation medium for 0, 11, or 30 days, and samples were collected for bulk or single-cell sequencing. At both transcriptomic and epigenomic level, those organoids recapitulate the temporal development of the human neocortex between 8 and 16 postconceptional week. Indeed, organoid system has been used in many studies to dissect temporal and spatial phenotypes in development. A decreased cell-cycle length was observed in early stage of organoids derived from ASD patients, but not in later stage (Mariani et al., 2015). In a separate study, an increased thickness of TBR1^+^ cortical regions was identified in ASD organoids (Schafer et al., 2019), which has been suggested to result from the increased NPC proliferation.

To better resemble the brain environment, CNS components that originate outside the ectoderm are generated and introduced to the brain organoids. In a study investigating neurite shaping and neuroinflammation, induced microglia from hPSCs have been introduced to organoid cultures (Abud et al., 2017). The neurovascular unit is crucial in CNS for supplying the brain with nutrients from blood stream and, although it is still missing in brain organoids, recent studies have successfully built functional blood vessels *in vitro* from hPSCs (Wimmer et al., 2019).

Early efforts to generate brain organoids from hPSCs largely depended on cell self-assembly with minimum external patterning (Lancaster et al., 2013). The “intrinsic protocol” offers the brain organoids more degrees of freedom to develop; however, it also makes the system less reliable in which the neural induction is less predictable, and a variety of brain region identities are randomly distributed in each individual organoid. Therefore, “directed approaches” have been developed to guide the organoids to acquire brain region-specific properties (Muguruma et al., 2015; Sakaguchi et al., 2015; Jo et al., 2016; Qian et al., 2016). In these protocols, hPSCs are first differentiated towards ectoderm and subsequently specified to the targeted brain regions by exogenous molecules (Di Lullo and Kriegstein, 2017). Dorsal forebrain is the default fate of the ectodermal tissues; therefore, minimal intervention is required to generate forebrain organoids. By manipulating pathways that are critical during development—for instance, Wnt, BMP, FGF, or SHH, CNS regions of ventral forebrain (Bagley et al., 2017; Birey et al., 2017), MGE (Xiang et al., 2017), midbrain (Jo et al., 2016; Qian et al., 2016; Xiang et al., 2017), hippocampus (Sakaguchi et al., 2015), hypothalamus (Qian et al., 2016), and cerebellum (Muguruma et al., 2015) can be induced in brain organoids (see Table 2 for the selected approaches). Intriguingly, many human brain-specific features are observed in brain region-specific organoids, which highlights the advantage of using organoid system to study human brain development and decode human-specific disorders. For instance, the human cerebral cortex, which is different from that in other species like mice, expands with folded surface (gyrencephalic) (Dehay et al., 2015). The complexification of the human cortex starts early in development and is associated with the emergence of the outer SVZ (oSVZ), particularly outer radial glia (oRG) (Dehay et al., 2015). In cortical organoids, the structure of the oSVZ has been observed (Qian et al., 2016; Bershteyn et al., 2017). Organoids derived from patients with nearly absent cortical folding, namely Miller-Dieker syndrome (MDS), were smaller in size and displayed deficits in precursor cleavage and early neuron migration (Iefremova et al., 2017). Consistently, in a separate study, mitotic delay in oRG was observed in MDS organoids (Bershteyn et al., 2017). On the other hand, the expansion and folding of the neuroepithelial structure are observed in PTEN mutant human cerebral organoids, which is linked to increased neural progenitor proliferation (Li et al., 2017).

**Table 2:** Select protocols for generation of brain organoids.

Reference	Organoid type	Cell lines	Patterning	Features
(Lancaster et al., 2013)	Whole brain	hESCs, hiPSCs	Minimum	Proof-of-concept study showing the feasibility of generating human brain organoids
(Quadrato et al., 2017)	Whole brain	hiPSCs	Minimum	Identification of diverse cell types in brain organoids by performing large-scale single-cell RNA-sequcing. Demonstratin of neuronal function by MEA analysis in long-term cultured brain organoids
(Li et al., 2017)	Whole brain	hESCs	Minimum	Induction of cortical expansion and folding in PTEN-deleted human brain organoids
(Eiraku et al., 2008)	Cortex	mESCs	Wnt inhibition, Nodal inhibition	Pioneer study for generation of serum-free suspension culture that can recapitulize the early corticogenesis
(Kadoshima et al., 2013)	Cortex	hESCs	Wnt inhibition, TGFβ inhibition, 40% O_2_	Generation of hESC-derived cortical tissue in long-term suspension culture to recapitulate second-trimester neocorticogenesis
(Bershteyn et al., 2017)	Cortex	hiPSCs	Wnt inhibition, TGFβ inhibition, 40% O_2_	Cortical organoids were used to model Miller-Dieker Sydrome, a severe type of lissencephaly
(Pasca et al., 2015)	Cortical spheroids	hiPSCs	Dual SMAD inhibition	Development of a simple method with no cell plating and embedding, allows long-term neuronal culture for study of functional human neurons and glial cells
(Sloan et al., 2017)	Cortical spheroids	hiPSCs	Dual SMAD inhibition	Demonstration of human astrocytes maturation captured in long-term cultured cortical spheroids
(Birey et al., 2017)	Dorsal and ventral forebrain	hiPSCs	Dual SMAD inhibition, Wnt inhibition and SHH activation	Generation of dorsal and vental forebrain organoid assembly to resemble the saltatory migration of interneuron in early forebrain development. Identified abnormal interneuron migration in organoid assembly derived from Timothy syndrome
(Xiang et al., 2017)	MGE	hESCs, hiPSCs	Dual SMAD inhibition, Wnt inhibition, followed by SHH activation	Fusion of cortical and MGE organoids to study human interneuron migration and integration
(Bagley et al., 2017)	Ventral forebrain	hESCs	Wnt inhibition and SHH activation	Demonstration of human interneuron migration from ventral to dorsal using organoids fusion. Potentially allows to analyze complex neurodevelopmental defects that might involve the interneuron abnormality
(Qian et al., 2016)	Dorsal forebrain	hESCs, hiPSCs	Dual SMAD inhibition	Generation of homogenious organoids for modeling embryonic cortical development. Model bisphenol A and ZIKV exposure during cortical neurogenesis
(Qian et al., 2016)	Midbrain	hiPSCs	Dual SMAD inhibition, SHH activation, GSK3β inhibition, FGF-8	N/A
(Qian et al., 2016)	Hypothalamus	hiPSCs	Dual SMAD inhibition, Thioglycerol	N/A
(Muguruma et al., 2015)	Cerebellum	hESCs	Insulin, FGF2, TGFβ inhibitor, FGF19, SDF1	Generation of functional 3D cerebellar culture for study of cerebellar ontogenesis
(Sakaguchi et al., 2015)	Hippocampus and choroid plexus	hESCs	Modulation of BMP and Wnt signaling	Pioneer study for generation of brain region specific 3D tissues, potentially useful for study of hippocampus-related disorders *in vitro*
(Jo et al., 2016)	Midbrain	hESCs	Dual SMAD inhibition, SHH, FGF-8, GSK3β inhibition	Generation of functional dopaminergic and neuromelanin-producing neurons in human midbrain organoids. Potentially can be used for study of Parkinson’s disease
(Madhavan et al., 2018)	Oligodendrocytes containing neurocortical spheroids	hiPSCs	Dual SMAD inhibition (D1-7), PDGF-AA and IGF-1 (D50-60), T3 and PERK inhibition (D60-70)	Generation of oligodendrocytes and myelin in neurocortical spheroids for study of cortical development. Test the effect of promyelination drugs and model Pelizaeus–Merzbacher disease

Human brain development requires that cells migrate long distances from one brain region to another (Pasca, 2019), and cellular processes extend out to connect different brain regions. The fusion of two or more region-specific organoids may enable the study of inter-regional interactions and circuit assembly in human CNS. Glutamatergic pyramidal neurons and GABAergic interneurons are two major neuronal types that compose the neuronal circuits in the cerebral cortex. While glutamatergic neurons are generated in dorsal forebrain, GABAergic interneurons are primarily born in the ventral forebrain and migrate over long distances to integrate into cortical circuits. Disruption in interneuron migration is linked to neurodevelopmental disorders such as ASD and epilepsy (Birey et al., 2017). To study interneuron migration, region-specific organoids resembling MGE and dorsal forebrain cortical organoids are generated (Birey et al., 2017; Xiang et al., 2017) and fused together. The migration of interneurons has been identified specifically from ventral forebrain organoids to dorsal forebrain cortical organoids, whereas the reverse is not observed. However, abnormal interneuron migration is observed in organoids derived from patients with Timothy syndrome (Birey et al., 2017).

Thus far, organoid models have been largely used to study events in early brain development, including neuroprogenitor proliferation (Qian et al., 2016), interneuron migration (Birey et al., 2017; Xiang et al., 2017), and formation of cortical layers (Qian et al., 2016; Schafer et al., 2019). To build a model system that resembles the human brain in postnatal stage requires that brain organoids survive for extended periods of time and remain healthy. Although brain organoids can be kept in culture for up to two years (Pasca et al., 2015), the overall survival rate is low and the necrotic center becomes prominent in long-term culture, which might be a concern when organoids are used to recapitulate disease phenotype in postnatal brain. Lack of vascular circulation is believed to be one major factor that limits the life span of brain organoids. To address this issue, brain organoids have been generated *in vitro* and transplanted into adult mouse brain (Mansour et al., 2018). Intriguingly, the grafted organoids gained functional vascularization system soon after transplantation, showing progressive neuronal maturation and gliogenesis, and connecting to host brains by sending out extensive axons. Importantly, organoids can survive as long as 200+ days in mouse brain. While this strategy presents great promise in long-term maintenance of brain organoids, the developmental stage of those organoids remains to be determined. Another question is whether human brain tissue can respond to visual, auditory, or motor stimuli that are presented to the host mouse.

Brain organoids present powerful potential for studying human brain development. Despite certain limitations in reproducibility, scalability, and long-term survival, this model system offers a unique opportunity to tackle questions that are challenging to address in other systems such as 2D culture system and rodents.

## CHALLENGES OF MODELING NEUROPSYCHIATRIC DISORDERS USING HIPSCS

Although the use of iPSCs to study neuropsychiatric disorders has provided unique insights to the field and has demonstrated great potentials to bridge the clinical features with cellular phenotypes that can be studied in the lab, there are a number of obstacles to be overcome. First of all, it has become increasingly clear that there is a great amount of variability in the reprogramming of hiPSCs from somatic cells and in the generation of brain cells from the hiPSCs. Variations may occur in any steps from reprogramming to differentiation. To reduce the technical variability and enable the identification of biological phenotypes, several approaches should be taken into consideration. Careful quality control is necessary at every step. For instance, only the iPSC clones that show full pluripotency should be selected for downstream experiments. Typically, a biological phenotype is ensured only if it’s observed in two or more iPSC clones from the same subject. Genetic background variations represent another challenge to iPSC disease modeling due to epistatic effects. Careful selection of control subjects with comparable age, gender, and genetic background is thus crucial to identifying disease-relevant phenotypes. Furthermore, although enormous attempts have been made to generate distinct neural cell types *in vitro*, it remains to be explored to what extent these iPSC-derived neural cells recapitulate the molecular and cellular signatures of their *in vivo* counterpart. In addition, many components of the building blocks of brains are still missing in an *in vitro* system, such as blood vessels, pericytes, and immune cells. These limitations should be considered and addressed when using hiPSC-derived cells to model human neuropsychiatric disorders.

## CONCLUSIONS

In conclusion, hiPSC-based disease modeling technology has demonstrated the potential to advance many aspects of biomedical research. We present here recent progress in the directed differentiation of neuronal subtypes, glial cells, and 3D brain organoid models, and the use of these models to study neuropsychiatric disorders. While many challenges remain to be solved before translating hiPSC-based discoveries directly into clinics, the opportunity to investigate specific neural subtypes using patient-specific cells, to study disease etiology in a 3D cellular model recapitulating features of human brain, and to establish a scalable screening platform for drug discovery *in vitro*, all support the idea that hiPSC-based research could present a new way to discover disease-relevant functional changes, a better understanding of disease etiology, and a new paradigm for neuropsychiatric drug discovery.
